# Dispensing by Family Pharmacists as a Potential Factor in Pharmacist-initiated Prescription Change: A Retrospective Observational Study

**DOI:** 10.2188/jea.JE20220165

**Published:** 2023-12-05

**Authors:** Takakiyo Nishikawa, Nobuo Sakata, Takehiro Sugiyama, Nanako Tamiya

**Affiliations:** 1 Department of Health Services Research, Faculty of Medicine, University of Tsukuba, Ibaraki, Japan; 2 Health Services Research and Development Center, University of Tsukuba, Ibaraki, Japan; 3 Diabetes and Metabolism Information Center, Research Institute, National Center for Global Health and Medicine, Tokyo, Japan; 4 Institute for Global Health Policy, Bureau of International Health Cooperation, National Center for Global Health and Medicine, Tokyo, Japan

**Keywords:** Japan, elderly, prescription change, pharmacist, polypharmacy

## Abstract

**Background:**

No studies in Japan have examined whether dispensing by family pharmacists, who are incentivized by reimbursement to provide continuous and exclusive medication management, results in prescription changes. Our primary objective was to identify the variables affecting prescription changes, particularly to investigate dispensing by family pharmacists as a possible factor.

**Methods:**

We identified 333,503 records of pharmacy claims data from patients aged 65 years or older who received medication instructions at outpatient pharmacies at Tsukuba, a medium-sized city near Tokyo, between April 2018 and March 2019. We extracted data on dispensing by family pharmacists, number of medicines, patient sex, patient age, and pharmacy category. A multilevel modified Poisson regression analysis was performed to analyze the correlation between dispensing by family pharmacists and pharmacist-initiated prescription change.

**Results:**

Dispensing by family pharmacists was 1.37 times more likely to involve a record of prescription change than dispensing by non-family pharmacists. Older age, female sex, polypharmacy, and small-scale pharmacies were also found to be factors.

**Conclusion:**

This study indicated that dispensing by family pharmacists was a potential factor for pharmacist-initiated prescription changes that may prevent excessive medication and limit pharmacological interactions. Since the likelihood of inappropriate prescriptions being issued varies from hospital to hospital, subsequent studies should take into account the quality of each institution.

## INTRODUCTION

Polypharmacy is a growing problem worldwide and is experienced by approximately 30% of elderly people in Japan.^1^ As people grow older, the number of drugs that they use increases.^2^ Pharmacists play a key role in limiting drug usage through an effort that involves collaborating with prescribers. The collaboration results in interventions, such as pharmaceutical management, which includes the assessment of dosages, proportional dosing, and drug interactions by the pharmacist. Several studies have revealed that pharmacist interventions prevent polypharmacy and improve medication adherence.^3^^–^^7^

In April 2016, the Japanese Ministry of Health, Labour and Welfare (MHLW) initiated a program of registered family pharmacists for patients, such that those pharmacists were empowered to handle continuous and exclusive medication management in cooperation with family physicians.^8^ According to MHLW, patients can feel free to consult with their family pharmacist about their medications. Patients can also expect to improve their medication status by having a family pharmacist who can help prevent multiple medications, duplicate medications, and drug interactions.^9^ Pharmacies can receive additional payments when family pharmacists provide service according to a number of strict requirements, including carefully collecting the patients’ medication and hospital visit history.^10^

A questionnaire survey conducted by the MHLW in 2017 determined that the average number of prescription changes initiated by pharmacists per month was higher at pharmacies with a family pharmacist than at those without.^11^ However, there was no statistical analysis accompanying this survey. In addition, the survey did not consider patient or medical institution factors, such as patient age, patient sex, number of drugs, and quality of pharmacies and hospitals. While this questionnaire survey focused on whether pharmacies had family pharmacists and compared the quality of services, there is a complete lack of studies concerning if and how family pharmacists actually dispense. Whether patients who are served by family pharmacists are more likely to experience pharmacist-initiated prescription changes that may prevent polypharmacy and harmful drug interactions than those receiving medications from non-family pharmacists is still unclear.

In this context, we hypothesized that patients who experience dispensing by family pharmacists receive more careful medication assessment than patients who are served by non-family pharmacists and, therefore, are more likely to have a record of pharmacist-initiated prescription changes that may prevent polypharmacy and drug interactions. We aimed to investigate the association between dispensing by family pharmacists and pharmacist-initiated prescription changes as revealed in pharmacy claims data.

## METHODS

### Setting

This was a retrospective observational study using pharmacy claims data from Tsukuba, a medium-sized city in the Tokyo metropolitan area in Japan, between April 2018 and March 2019. The city population was approximately 250,000; approximately 20.0% were aged 65 years or older as of July 2022. In Japan, 76% of outpatient care is prescribed outside hospitals and clinics; therefore, most patients receive their medication at dispensing pharmacies.

Japan’s medical and dispensing fees are unified throughout the country, and the country has a universal health insurance system. Dispensing by family pharmacists is part of a reimbursement program in which pharmacists provide continuous and exclusive medication management in cooperation with family physicians. When a patient registers with a family pharmacist, the patient’s medication should be allocated by the same family pharmacist that the patient registers with. Furthermore, the family pharmacist is expected to obtain information on all medical institutions that the patient visits as well as all prescription drugs, over-the-counter drugs, and health foods that the patient consumes. The family pharmacist is also responsible for recording information on the patient’s medication history.

### Data source

The pharmacy claims data obtained was restricted to people enrolled with the National Health Insurance and Late-Stage Medical Care System for the Elderly in Tsukuba. The National Health Insurance insures mainly self-employed and unemployed individuals aged ≤74 years. The Late-Stage Medical Care System for the Elderly is a service for citizens aged 75 and over. Pharmacy claims data of people who joined other insurance systems, such as employment-based health insurance, were not included in the data reviewed.^12^^,^^13^ The available data included dosage of medication, date of administration, and services provided by the pharmacy, such as package delivery and referral to medical institutions. A unique number was assigned to each pharmacy visit to identify the dispensing details.

### Eligibility

Records of pharmacy claims data that contained the basic fee for receiving prescriptions at pharmacies from April 2018 to March 2019 were included in this study. Japan’s dispensing fees are renewed every 2 years, in April of even-numbered years. In order to conduct the analysis based on the most recent reimbursement program among the available data, the study period was set from April 2018 to March 2019.

**Figure 1.**
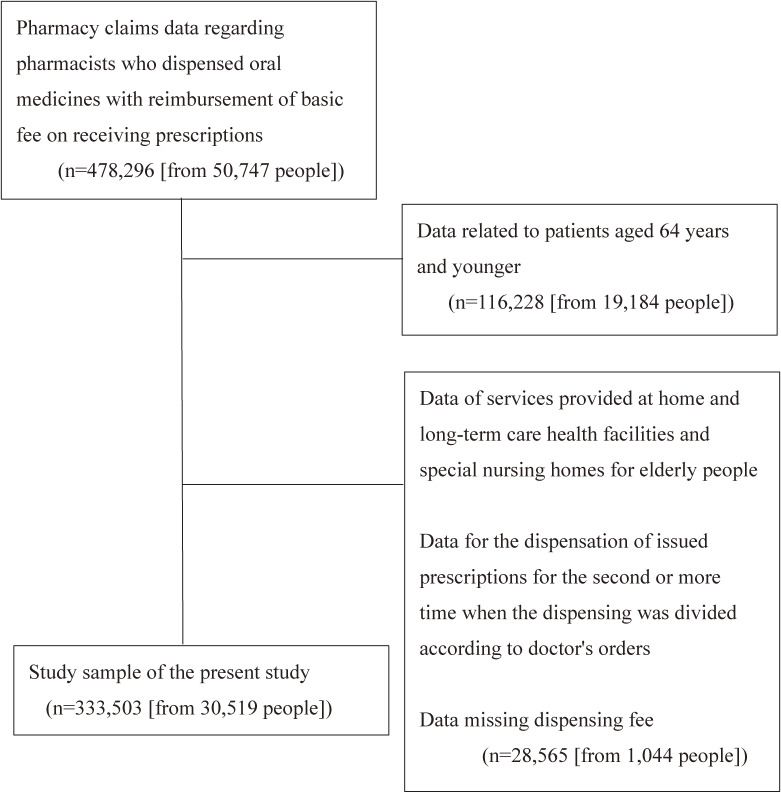
Flow chart of study sample selection

Figure 1 shows the sample selection process. We extracted pharmacy claims data corresponding to events in which pharmacists dispensed oral medicines for patients aged ≥65 years. To ensure comparability with several previous studies on polypharmacy in Japan, this study included people aged 65 years and older.^14^^,^^15^ Additionally, the age limit of this study’s population was in line with the World Health Organization’s (WHO) note that people aged 65 years and older are a vulnerable group at risk of polypharmacy.^16^ If dispensing was divided into two or more times according to the doctor's order, we included data from the first instance only.

We excluded data for people who received home-based medical care and/or lived in long-term care health facilities and special nursing homes for elderly people. This is because these patients often receive other services, such as home drug guidance, and are different from general outpatients. We also excluded data where the basic fee records were missing.

### Outcome

The outcome variable was prescription change. We defined prescription change as follows: a change in a prescription resulting from inquiries made by the pharmacist to the prescribing physician based on the assessment of the patient’s medication history for the purpose of preventing duplicate dosing and drug interactions. Pharmacies can receive an additional fee for preventing polypharmacy and drug interactions (AFPPD) when pharmacists initiate a prescription change and an AFPPD is recorded in the claims data. Therefore, we treated the AFPPD as a surrogate for prescription change. The requirements of the AFPPD are described in eTable 1.

### Primary explanatory variable and covariates

The primary explanatory variable was dispensing by family pharmacists. Dispensing by family pharmacists was recorded in the data as “fee for family pharmacist assessment”, which represented remuneration for a registered family pharmacist to assess the patient’s medication, obtain information on all medical institutions that the patient visits, all prescription drugs, over-the-counter drugs, and health foods that the patient consumes, and record information on the patient’s medication history (eTable 1). The covariates were patient age (three categories: 65–74, 75–84, and ≥85 years), patient sex, number of drugs (two categories: ≤4 and ≥5), and pharmacy category (5 categories). The number of drugs was counted for each drug that was assigned a code by MHLW. We defined polypharmacy as use of five or more oral drugs in accordance with previous studies and the WHO definition.^14^^,^^16^^,^^17^ To ensure comparability with several previous studies on polypharmacy, this study included only oral drugs.^15^^,^^17^

The categorization of pharmacies into five types, which was distinguished in the reimbursement program in Japan, was based on the number of prescriptions that a pharmacy received and the strength of its relationship with a particular hospital or clinic. Roughly, small-scale pharmacies that tended to accept prescriptions from various medical institutions were classified as category 1; middle-scale pharmacies that accepted a high percentage of prescriptions from a specific medical institution were classified as category 2; relatively large-scale pharmacies that accepted a high percentage of prescriptions from a specific medical institution were classified as category 3; large-scale pharmacies that accepted a high percentage of prescriptions from a specific medical institution were classified as category 4; and pharmacies that had real estate transactions with a particular hospital or clinic and accepted a high percentage of prescriptions from a specific medical institution were classified as category 5 (eTable 1). We considered the categorization of pharmacies as a possible confounding factor and considered it as a covariate because large-scale pharmacies may have a better training system, and pharmacies that are more dependent on a particular medical institution tend to prescribe specific drugs and may be less aware of drug interactions.

### Statistical analyses

A descriptive analysis of pharmacy visits was performed. The unit of analysis was pharmacy visit, stratified on the basis of dispensing by family pharmacists. Bivariate analysis was performed using multilevel modified Poisson regression analysis to determine the association between the primary explanatory variable (dispensing by family pharmacists) and prescription change.^18^ All the independent variables used in the analysis were categorical or dichotomous. Multivariable analysis was performed using a multilevel modified Poisson regression. This analysis was performed to determine possible determinant factors of prescription change, with participants at level 1, claims data from pharmacies on a monthly basis at level 2, and pharmacy visits at level 3.

It should be noted that registration with a family pharmacist did not mean service would always be received from that pharmacist. This is because even patients who had family pharmacists could visit pharmacies other than the pharmacy at which they had registered with their family pharmacist. Therefore, in this analysis, we compared the data of dispensing by family pharmacist and the data of dispensing by non-family pharmacist using pharmacy visit as the unit of analysis. We list patient characteristics in Table 1, focusing on registrations with family pharmacists. The analyses in Table 2 and Table 3 were performed on a per pharmacy visit basis. To identify heterogeneity, we conducted subgroup analyses by age group and sex.

**Table 1.** Patient characteristics

	Patients who registered with family pharmacists^a^ *n* = 1,386	Patients who did not register with family pharmacists *n* = 29,133	*P*
Patient’s age, years, *n* (%)^b^			
65–74	451 (32.6)	14,268 (49.0)	<0.001
75–84	652 (47.1)	10,529 (36.1)	
≥85	283 (20.4)	4,336 (14.9)	
Sex of patient, *n* (%)			
Male	603 (43.5)	12,951 (44.5)	
Number of drugs, *n* (%)^c^			
Five or more	676 (48.8)	8109 (27.8)	<0.001
Category of pharmacy, *n* (%)^d^			
Category 1	366 (26.4)	19,927 (68.4)	<0.001
Category 2	98 (7.1)	639 (2.2)	
Category 3	12 (0.9)	2,442 (8.4)	
Category 4	910 (65.7)	6,028 (20.7)	
Category 5	0 (0.0)	97 (0.3)	

^a^Patients who were dispensed medication by a family pharmacist at the first service during the observation period.

^b^Age at the first service from pharmacists during the observation period.

^c^Number of drugs at the first service from pharmacists during the observation period.

^d^Category of pharmacy at the first service from pharmacists during the observation period.

**Table 2.** Pharmacy visit characteristics

	Dispensing by family pharmacists *n* = 16,148	Dispensing by non-family pharmacists *n* = 317,355	*P*
Patient’s age, years, *n* (%)			
65–74	4,512 (27.9)	132,715 (41.8)	<0.001
75–84	7,871 (48.7)	129,385 (40.8)	
≥85	3,765 (23.3)	55,255 (17.4)	
Sex of patient, *n* (%)			
Male	6,706 (41.5)	140,109 (44.1)	<0.001
Number of drugs, *n* (%)			
Five or more	7,723 (47.8)	100,994 (31.8)	<0.001
Category of pharmacy, *n* (%)			
Category 1	5,350 (33.1)	223,251 (70.3)	<0.001
Category 2	1,124 (7.0)	5,952 (1.9)	
Category 3	230 (1.4)	28,044 (8.8)	
Category 4	9,444 (58.5)	58,118 (18.3)	
Category 5	0 (0.0)	1,990 (0.6)	

**Table 3.** Factors associated with prescription change

	**Bivariate modified Poisson regression**					**Multivariable modified Poisson regression**		

		Prescription change			Likelihood ratio test	Prescription change		Likelihood ratio test
	
	%	Incidence proportion (%)	Incidence proportion ratio	(95% CI)	*P*	Adjusted incidence proportion ratio	(95% CI)	*P*
Dispensing by family pharmacists								
No (*n* = 317,355)	95.2	1.08	(reference)			(reference)		
Yes (*n* = 16,148)	4.8	1.91	1.63	(1.42–1.87)	<0.001	1.37	(1.19–1.59)	<0.001

^*^Patient age, patient sex, number of drugs, and category of pharmacy were considered as covariates (eTable 3).

All analyses were performed using Stata version 15 (StataCorp, College Station, TX, USA). Statistical significance was set at *P* < 0.05. The assumptions for the analysis were assessed during the analysis.

This study was approved by the Ethics Committee of the University of Tsukuba (approval number: 1445). The data utilized for this study were anonymous, and individual consent was not obtained. All the data had been stripped of the names and other personal information, and unique IDs were assigned to link multiple data before they were received from Tsukuba.

## RESULTS

### Patient and pharmacy visit characteristics

We present the patient characteristics in Table 1. In the present study, 30,519 people were included in the database analysis. Of the 30,519 people included in the study, 1,386 were patients who registered with family pharmacists, and 29,133 were patients who did not. Moreover, 67.5% of patients who registered with family pharmacists were aged 75 years and older, whereas 51.0% of patients who did not were aged 75 years and older. Half of the patients who registered with family pharmacists (48.8%) and less than one-third of patients who did not (27.8%) took five or more types of drugs.

Table 2 lists the pharmacy visit characteristics. Of the 333,503 pharmacy visits included in the study, 16,148 visits involved dispensing by family pharmacists and 317,355 involved dispensing by non-family pharmacists. Furthermore, for patients aged 75 years and older, 72.0% of visits involved dispensing by family pharmacists, whereas 58.2% of visits involved dispensing by non-family pharmacists. The percentages of visits involving dispensing by family pharmacists and dispensing by non-family pharmacists with use of five or more types of drugs were 47.8% and 31.8%, respectively. Moreover, 33.1% of visits involving dispensing by family pharmacists were at category 1 pharmacies. In contrast, 70.3% of visits involving dispensing by non-family pharmacists were at category 1 pharmacies.

We also clarified the pharmacy visit characteristics by pharmacy category. Category 1 pharmacies featured the highest proportion (68.5%) of pharmacy visits. The use of five or more types of drugs was recorded in 24.2% of visits to category 5 pharmacies and 35.5% of visits to category 4 pharmacies. Moreover, 15.9% of visits involved dispensing by family pharmacists at category 2 pharmacies, and 2.3% of visits involved dispensing by family pharmacists at category 1 pharmacies. The percentage of prescription change ranged from 0.3% (category 3) to 1.4% (category 4) (eTable 2).

### Prescription changes to prevent excessive medication and interactions

We also clarified the pharmacy visit characteristics by prescription changes. Of the 333,503 pharmacy visits included in the study, 3,752 resulted in prescription change being recorded. The incidence proportion of prescription change was 1.13%. The incidence proportions of prescription change were 1.08% and 1.91% in the non-family-pharmacist-dispensed and family-pharmacist-dispensed groups, respectively. Additionally, 21.9% of the prescription-change group comprised patients aged 85 years or older. In contrast, 17.6% of the non-prescription-change group comprised patients aged 85 years or older. The proportions of pharmacy visits recording five or more types of drugs were 46.9% and 32.4% in the prescription-change group and non-prescription-change group, respectively.

### Factors of prescription change

Table 3 shows the association between potential factors and prescription changes, which were obtained from bivariate and multivariable modified Poisson regression analyses.

#### Bivariate modified Poisson regression

Dispensing by family pharmacists was 1.63 times more likely to involve prescription change compared to dispensing by non-family pharmacists (incidence proportion ratio [IPR] 1.63; 95% confidence interval [CI], 1.42–1.87). The possibility of prescription change was 1.27 times higher for people aged 75–84 years (IPR 1.27; 95% CI, 1.16–1.39) and 1.49 times higher for people aged 85 years or older (IPR 1.49; 95% CI, 1.34–1.66) than that for people aged 65–74 years. The possibility of prescription change was 1.79 times higher for pharmacy visits listing five or more types of drugs than for visits where four or fewer types of drugs were noted (IPR 1.79; 95% CI, 1.67–1.93). In addition, the possibility of prescription change was 0.27 times lower for pharmacies in category 3 than for pharmacies in category 1 (IPR 0.27; 95% CI, 0.21–0.33) (eTable 3).

#### Multivariable modified Poisson regression

The service of prescription change was 1.37 times more likely to have been recorded by family pharmacists than by non-family pharmacists (IPR 1.37; 95% CI, 1.19–1.59). The possibility of prescription change was 1.20 times higher for pharmacy visits by people aged 75–84 years (IPR 1.20; 95% CI, 1.10–1.31) and 1.29 times higher for pharmacy visits by people aged 85 years or older (IPR 1.29; 95% CI, 1.16–1.44) than that for visits by people aged 65–74 years. The possibility of prescription change was 1.70 times higher for pharmacy visits recording five or more types of drugs than for those recording four or fewer types of drugs (IPR 1.70; 95% CI, 1.58–1.84). In addition, the possibility of prescription change was 0.27 times lower for visits at category 3 pharmacies than for visits at category 1 pharmacies (IPR 0.27; 95% CI, 0.21–0.33) (eTable 3).

Subgroup analyses by age group and sex showed similar results to the main analysis. Family pharmacists were more likely to have recorded prescription changes than non-family pharmacists in all subgroup analyses, except for the group aged 85 years or older (eTable 4 and eTable 5). We also examined the heterogeneity by including the interaction terms. In the analyses, the interaction terms were not statistically significant.

## DISCUSSION

This study demonstrated that dispensing by family pharmacists was a potential factor for prescription changes that may prevent excessive medication and unwanted drug interactions. To our knowledge, this is the first study to illustrate the potential effects of dispensing by family pharmacists.

In this study, the association between prescription change and dispensing by family pharmacists was statistically significant in the multivariable analysis. One possible reason for this result is that family pharmacists are required to provide higher-quality medication assessment. To receive an incentive payment for family pharmacist assessment, pharmacies are required to keep track of all the medications and hospital visits of family pharmacists’ patients. In addition, pharmacies that meet certain criteria for providing family pharmacist service need to assign pharmacists with a certification for continuing professional development.^9^ Based on the patients’ detailed information and their own updated knowledge, family pharmacists are more likely to perceive polypharmacy or inappropriate medication of patients.

In this study, dispensing by family pharmacists was found to be higher among elderly and polypharmacy patients. Past studies have shown that elderly people and those with polypharmacy are more likely to take inappropriate medications.^19^^–^^22^ It may be possible that pharmacists recognize that the elderly and those with polypharmacy are more likely to take inappropriate medications and, therefore, encourage them to register with family pharmacists.

This study found that the increasing number of drugs was one of the factors associated with prescription change, which is consistent with other results.^19^^,^^21^^,^^22^ Previous cross-sectional and retrospective cohort studies have shown that patients taking more drugs are more likely to receive potentially inappropriate prescriptions than patients taking fewer drugs.^19^^,^^21^^,^^22^ A similar finding can be observed for age. The present study showed that older age may be a factor in prescription change. A previous cohort study has revealed that the older people were, the more likely they were to receive potentially inappropriate prescriptions.^20^ Combined with the results of our study, it appears that intervention by a robust family pharmacist for older patients taking many drugs may prevent adverse events due to excessive medication use and drug interactions.

The crude value of the absolute difference in prescription change based on the presence or absence of a family pharmacist was 0.83%. We consider this difference to be clinically significant given the negative impact of medication errors. However, future research is needed to determine whether the cost is justified, taking into account the other benefits of having a family pharmacist.

Several limitations of this study should be considered when interpreting its findings. First, the study was based on a single city in Japan; therefore, generalizability to other areas in Japan might be limited. Second, we did not consider the patients’ diseases. It is possible that there is a group of diseases for which prescription changes are more likely to occur, in which case they were not adjusted for in this study. Third, although there are reimbursement requirements for family pharmacists to provide higher quality medication assessment than non-family pharmacists, it is not possible to determine from the claims data whether they actually implement all the requirements. Therefore, it is not possible to know with certainty whether having a family pharmacist indeed results in changing interventions and thus preventing inappropriate medication use. In addition, this study did not address the likelihood that the incidence of inappropriate prescriptions might vary from hospital to hospital. Further investigation that includes an assessment of the quality of associated institutions is warranted. This is made more obvious upon recognizing that our pharmacy categorization approach combined numbers of prescriptions and organizational relationships, making it almost impossible to detect differences due to each of these factors independently.

In conclusion, dispensing by family pharmacists is associated with prescription changes that may prevent duplicate medications and adverse events due to drug interaction. In particular, elderly patients and patients taking multiple medications may benefit from this family pharmacist system through a reduction in the number of adverse events caused by prescriptions.

## SUPPLEMENTARY MATERIAL

eTable 1. Description of terminology

eTable 2. Pharmacy visit characteristics by category of pharmacy

eTable 3. Factors associated with prescription change

eTable 4. Results of multivariable modified Poisson regression analyses, stratified by age group

eTable 5. Results of multivariable modified Poisson regression analyses, stratified by sex

eTable 6. Codes in the reimbursement system
