# Supplementary material for: Dispensing by Family Pharmacists as a Potential Factor in Pharmacist-initiated Prescription Change: A Retrospective Observational Study
Source: J Epidemiol. 2023 Dec 5;33(12):618–23. doi: 10.2188/jea.JE20220165 (PMC10635808; doi:10.2188/jea.JE20220165)
Supplement: Supplementary file 1 [file je-33-618-s001.pdf]

eTable 1. Description of terminology

Terminology	Requirement
Fee for family pharmacist assessment	In addition to basic services, the following services are required to be provided.<br>– A certain pharmacist selected by the patient provides medical consultation to the patient based on an integrated and continuous understanding of the patient's medication status in cooperation with the physician.<br>– Medication guidance and other services for patients should be provided by the pharmacist.<br>– Information on all insurance medical institutions that the patient visits should be recorded, and all prescription medicines, over-the-counter drugs, and health foods that the patient consumes should be recorded.<br>– A system should be in place to provide 24-h consultation for patients.
Additional fee for preventing for polypharmacy and drug interactions (AFPPD)	The following services are required to be provided for additional fee.<br>– Based on the medication history or patient's information, pharmacists confirm the prescription with the prescribing physician.<br>– Pharmacists implement prescription change, including dose adjustment, based on interactions with concomitant medications or foods.

Category of pharmacy	Requirement
Category 1	Not falling under any of the following categories
Category 2	Not falling under any of the following categories<br>and<br>1) Number of prescription record per month (NP) >4,000 (more than 70% is from a particular hospital or clinic)<br>or<br>2) NP >2,000 (more than 85% is from a particular hospital or clinic)<br>or

	3) NP >4,000 from a particular hospital or clinic<br>or<br>4) NP >4,000 from hospitals or clinics in the same building with the pharmacy<br>or<br>5) NP >4,000 (if a certain pharmacy and other pharmacy are belonging to the same group company and they received highest percentage of NP from a same hospital or clinic, NP is total prescriptions that both pharmacies receive from the hospital or clinic)
Category 3	Not falling under any of the following categories<br>and<br>1) 400,000 ≥ NP >40,000 (total prescriptions of group company)<br>and<br>2)-1 More than 85% of NP is from a particular hospital or clinic<br>or<br>2)-2 Real estate transaction with a particular hospital or clinic
Category 4	Not falling category 5<br>and<br>1) NP >400,000 (total prescriptions of group company)<br>and<br>2)-1 More than 85% of NP is from a particular hospital or clinic<br>or<br>2)-2 Real estate transaction with a particular hospital or clinic
Category 5	1) Real estate transaction with a particular hospital or clinic<br>and<br>2) More than 95% of NP is from a particular hospital or clinic

AFPPD, additional fee for preventing for polypharmacy and drug interactions; NP, number of prescription records per month.

eTable 2. Pharmacy visit characteristics by category of pharmacy

	Category 1 n=228,601	Category 2 n=7,076	Category 3 n=28,274	Category 4 n=67,562	Category 5 n=1,990
Patient age, years, n (%)					
65–74	96,688 (42.3)	3,337 (47.2)	10,186 (36.0)	26,386 (39.1)	630 (31.7)
75–84	92,005 (40.2)	2,878 (40.7)	12,664 (44.8)	28,879 (42.7)	830 (41.7)
≥85	39,908 (17.5)	861 (12.2)	5,424 (19.2)	12,297 (18.2)	530 (26.6)
Sex of patient, n (%)					
Male	100,900 (44.1)	3,648 (51.6)	11,336 (40.1)	29,890 (44.2)	1,041 (52.3)
Number of drugs, n (%)					
Five or more	74,044 (32.4)	2,460 (34.8)	7,776 (27.5)	23,956 (35.5)	481 (24.2)
Pharmacy visits with family pharmacists dispensing, n (%)					
Yes	5,350 (2.3)	1,124 (15.9)	230 (0.8)	9,444 (14.0)	0 (0.0)
Prescription change, n (%)					
Yes	2,632 (1.2)	92 (1.3)	89 (0.3)	915(1.4)	24 (1.2)

eTable 3. Factors associated with prescription change

	Bivariate modified Poisson regression				Multivariable modified Poisson regression		
	Prescription change			Likelihood ratio test	Prescription change		Likelihood ratio test
	%	IP (%)	IPR	(95% CI)	*P*	Adjusted IPR	(95% CI)	*P*

	%	IP (%)	IPR	(95% CI)	*P*	Adjusted IPR	(95% CI)	*P*
Dispensing by family pharmacists								
No (n=317,355)	95.2	1.08	(ref)			(ref)		
Yes (n=16,148)	4.8	1.91	1.63	(1.42 – 1.87)	<0.001	1.37	(1.19 – 1.59)	<0.001
Patient age, years								
65–74 (n=137,227)	41.1	0.95	(ref)			(ref)		
75–84 (n=137,256)	41.2	1.19	1.27	(1.16 – 1.39)	<0.001	1.20	(1.10 – 1.31)	<0.001
≥85 (n=59,020)	17.7	1.39	1.49	(1.34 – 1.66)	<0.001	1.29	(1.16 – 1.44)	<0.001
Patient sex								
Male (n=146,815)	44.0	1.05	(ref)			(ref)		
Female (n=186,688)	56.0	1.18	1.13	(1.04 – 1.22)	<0.005	1.12	(1.03 – 1.21)	<0.01
Number of drugs								
Four or fewer (n=224,786)	67.4	0.89	(ref)			(ref)		
Five or more (n=108,717)	32.6	1.62	1.79	(1.67 – 1.93)	<0.001	1.70	(1.58 – 1.84)	<0.001

Category of pharmacy								
Category 1 (n=228,601)	68.5	1.15	(ref)			(ref)		
Category 2 (n=7,076)	2.1	1.30	1.07	(0.84 – 1.35)		1.03	(0.81 – 1.31)	
Category 3 (n=28,274)	8.5	0.31	0.27	(0.21 – 0.33)	<0.001	0.27	(0.21 – 0.33)	<0.001
Category 4 (n=67,562)	20.3	1.35	1.08	(0.99 – 1.19)		1.02	(0.92 – 1.12)	
Category 5 (n=1,990)	0.6	1.20	0.97	(0.61 – 1.54)		1.00	(0.63 – 1.59)	

CI, confidence interval; IP, incidence proportion; IPR, incidence proportion ratio; ref, reference

eTable 4. Results of multivariable modified Poisson regression analyses, stratified by age group

	Patient age 65–74 years			Patient age 75–84 years			Patient age ≥85 years		
	Prescription change		Likelihood ratio test	Prescription change		Likelihood ratio test	Prescription change		Likelihood ratio test
	Adjusted IPR	(95% CI)	*P*	Adjusted IPR	(95% CI)	*P*	Adjusted IPR	(95% CI)	*P*
Dispensing by family pharmacists									
No	(ref)			(ref)			(ref)		
Yes	1.69	(1.26 – 2.26)	<0.001	1.27	(1.05 – 1.55)	<0.05	1.24	(0.93 – 1.66)	
Patient sex									
Male	(ref)			(ref)			(ref)		
Female	1.25	(1.09 – 1.43)	<0.005	1.08	(0.96 – 1.22)		1.02	(0.85 – 1.21)	
Number of drugs									
Four or fewer	(ref)			(ref)			(ref)		
Five or more	2.03	(1.79 – 2.31)	<0.001	1.66	(1.48 – 1.85)	<0.001	1.41	(1.20 – 1.65)	<0.001
Category of pharmacy									
Category 1	(ref)			(ref)			(ref)		

Category 2	0.92	(0.62 – 1.36)		1.11	(0.78 – 1.57)		0.99	(0.54 – 1.81)	
Category 3	0.28	(0.18 – 0.42)	<0.001	0.26	(0.18 – 0.36)	<0.001	0.27	(0.17 – 0.43)	<0.001
Category 4	0.81	(0.68 – 0.96)	<0.05	1.13	(0.99 – 1.30)		1.14	(0.93 – 1.39)	
Category 5	1.33	(0.57 – 3.11)		0.82	(0.41 – 1.62)		0.93	(0.44 – 1.96)	

CI, confidence interval; IPR, incidence proportion ratio; ref, reference.

eTable 5. Results of multivariable modified Poisson regression analyses, stratified by sex

	Male			Female		
	Prescription change		Likelihood ratio test	Prescription change		Likelihood ratio test
	Adjusted IPR	(95% CI)	*P*	Adjusted IPR	(95% CI)	*P*
Dispensing by family pharmacists						
No	(ref)			(ref)		
Yes	1.47	(1.17 – 1.84)	<0.005	1.31	(1.09 – 1.58)	<0.005
Patient age, years						
65–74	(ref)			(ref)		
75–84	1.30	(1.13 – 1.49)	<0.001	1.13	(1.01 – 1.27)	<0.05
≥85	1.47	(1.22 – 1.75)	<0.001	1.20	(1.05 – 1.38)	<0.01
Number of drugs						
Four or fewer	(ref)			(ref)		
Five or more	1.78	(1.58 – 2.00)	<0.001	1.66	(1.50 – 1.83)	<0.001
Category of pharmacy						
Category 1	(ref)			(ref)		
Category 2	0.90	(0.63 – 1.28)		1.14	(0.82 – 1.59)	
Category 3	0.20	(0.13 – 0.30)	<0.001	0.31	(0.23 – 0.41)	<0.001
Category 4	0.88	(0.75 – 1.03)		1.12	(0.99 – 1.26)	
Category 5	1.09	(0.56 – 2.14)		0.91	(0.50 – 1.65)	

CI, confidence interval; IPR, incidence proportion ratio; ref, reference.

eTable 6. Codes in the reimbursement system

Services for fee	Codes
basic fee for category 1	410004110, 410004910
basic fee for category 2	410004210, 410005010
basic fee for category 3	410004710, 410005110
basic fee for category 4	410004810, 410005210
basic fee for category 5	410004610, 410005310
fee for family pharmacist assessment	440004010
fee for pharmacist assessment	440006710, 440006810, 440006910, 440007010, 440007110, 440007210
AFPPD	440005170, 440005270, 440005470, 440005570

AFPPD, additional fee for preventing for polypharmacy and drug interactions.
